# Optical coherence tomography angiography metrics in different stages of diabetic macular edema

**DOI:** 10.1186/s40662-022-00286-2

**Published:** 2022-04-05

**Authors:** Ruyi Han, Ruowen Gong, Wei Liu, Gezhi Xu

**Affiliations:** 1 Eye Institute and Department of Ophthalmology, Eye and ENT Hospital of Fudan University, 83 Fenyang Road, Shanghai, 200031 People’s Republic of China; 2 Key Laboratory of Visual Impairment and Restoration, Fudan University, Shanghai, People’s Republic of China; 3 Key Laboratory of Myopia (Fudan University), Chinese Academy of Medical Sciences, National Health Commission, Shanghai, People’s Republic of China

**Keywords:** Optical coherence tomography angiography, Diabetic macular edema, Vessel density, Acircularity index

## Abstract

**Background:**

To investigate the optical coherence tomography angiography (OCTA) characteristics of diabetic macular edema (DME) at different stages.

**Methods:**

This study was a cross-sectional study. Patients diagnosed with DME were recruited. DME was classified into early, advanced, and severe DME. The vessel density (VD) in the superficial vascular plexus (SVP), deep vascular plexus (DVP) and foveal avascular zone (FAZ) parameters, including FAZ area, FAZ perimeter, acircularity index and foveal VD in a 300-μm-wide region around the FAZ (FD-300), were calculated by the AngioVue software. A multivariate generalized estimating equation was used to evaluate the associations between visual acuity and OCTA metrics.

**Results:**

Ninety-two eyes from 74 patients with DME were included in this study. Compared to early (*P* = 0.006) and advanced DME (*P* = 0.003), the acircularity index was higher in severe DME. Both whole and parafoveal VD in the DVP decreased in eyes with severe DME compared to early DME (*P* = 0.018, *P* = 0.005, respectively) and advanced DME (*P* = 0.035, *P* = 0.012, respectively). In the multivariate generalized estimating equation, DME severity, FAZ area and foveal thickness were positively associated with worse visual acuity (*P* = 0.001, *P* = 0.007 and *P* = 0.001, respectively).

**Conclusion:**

Compared to early and advanced DME, severe DME showed increased irregularity in the FAZ and more extensive vessel damage in the DVP. Greater severity level of DME, larger FAZ area, and increased foveal thickness could be risk factors for poor visual acuity.

*Trial registration* The protocol was published in the Chinese Clinical Trial Registry (ChiCTR2000033082).

## Background

Diabetic retinopathy (DR) is rising dramatically due to the steep increase in global diabetes incidence. It has been estimated that the number of patients with DR is 103 million in 2020 and could increase to 161 million in 2045 [1]. As one of the leading causes of visual impairment and blindness in the working-age population and the elderly [2, 3], diabetic macular edema (DME), which can occur at any stage of DR, has attracted great attention.

Optical coherence tomography angiography (OCTA) visualizes different retinal vascular layers noninvasively and has been utilized to study the associations between vessel density (VD) and foveal avascular zone (FAZ) parameters and DME [4, 5]. However, these studies have focused on either the association between intravitreal injection and OCTA parameters or the comparison of OCTA parameters in DR with or without DME. The association between the stages of DME and OCTA parameters has rarely been reported.

In the past, DME severity was assessed via ophthalmoscopy or color fundus photography [6]. Currently, DME grading classifications are also based on spectral domain optical coherence tomography (SD-OCT) and fluorescein angiography [7–9]. Despite attempts to classify DME through a variety of methods, grading DME with more comprehensive details visible on SD-OCT could be more conducive to improving the understanding of DME pathophysiology. An international SD-OCT-based classification of DME called “TCED-HFV”, which considers the specific morphologic features of OCT, including foveal thickness, intraretinal cysts, the state of the ellipsoid zone (EZ) and the external limiting membrane (ELM), the presence of disorganization of the inner retinal layers (DRIL) , the quantity of hyperreflective foci, the occurrence of subfoveal fluid, and the vitreoretinal relationship has been proposed recently [10]. Thus, it is important to utilize the newly proposed grading [10] to evaluate DME severity and to further analyze the OCTA characteristics among different grades of DME.

The objective of this study was to evaluate DME severity and to analyze the association between DME severity and OCTA parameters. The association between OCTA metrics and visual acuity in DME was also studied.

## Methods

### Subjects

Between June 2019 and September 2021, patients with DME were recruited consecutively at the Eye and ENT Hospital of Fudan University. The protocol was published in the Chinese Clinical Trial Registry (ChiCTR2000033082). This cross-sectional observational study was approved by the Ethics Committee of the Eye and ENT Hospital of Fudan University and conformed to the tenets of the Declaration of Helsinki. All participants provided written informed consent.

Criteria for inclusion included a central retinal thickness (CRT) greater than 320 μm for males or 305 μm for females [11], a diagnosis of DME, and age ≥ 18 years old. In this study, SD-OCT (Spectralis, Heidelberg, Germany) was used to calculate CRT and evaluate DME, and DME was equivalent to center-involved DME, which was defined as intraretinal cystoid changes and/or retinal thickening and/or neurosensory retinal detachment involving the central ring of the ETDRS macular map [10]. Participants were excluded due to (1) OCTA images scan quality score less than 6/10, motion artifacts, inaccurate segmentation, blurry images, and poor centration; (2) macular atrophy, high myopia, uveitis, glaucoma, severe media opacities, and previous ocular trauma; or (3) laser photocoagulations, intravitreal drug application and ocular surgery in the 6-month period before the study.

Ophthalmic examinations including best-corrected visual acuity (BCVA; logMAR visual acuity), intraocular pressure (non-contact tonometer), slit-lamp biomicroscopy, color fundus photography, OCT and OCTA were performed. The evaluation of DME severity was based on an OCT-based grading protocol [10], and DME was classified into early, advanced, and severe DME. Early DME was characterized by the presence of small intraretinal cysts, well-recognizable and detectable inner retinal layers, EZ, and ELM, and increase in CRT less than 30% of upper normal values. Advanced and severe DME were both defined by a CRT above 30% of upper normal values, macrocysts and/or multiple intraretinal cystoid spaces, and/or the loss of clear demarcation in inner retinal layers. The EZ/ELM may be impaired but still partially visible in the fovea in advanced DME, while the EZ/ELM are mostly undetectable in severe DME. DR was classified as non-proliferative (NPDR) or proliferative DR (PDR) according to the ETDRS Retinopathy Severity Scale [12]. Glycated hemoglobin A1c, serum lipids, and blood pressure were collected within two weeks of the start of the study. Hypertension was defined as a self-reported history of hypertension and/or a clinic blood pressure ≥ 140/90 mmHg [13]. Increased total cholesterol (≥ 5.2 mmol/L), low-density lipoprotein cholesterol (≥ 3.35 mmol/L), and triglyceride levels (≥ 2.25 mmol/L) were regarded as hyperlipidemia [14, 15]. Other relevant data, including age, sex, body mass index, duration of diabetes, type of diabetes, renal impairment, history of medication, and smoking and drinking status, were also recorded.

### OCTA

OCTA images were acquired after pupillary dilation using AngioVue OCTA (RTVue XR Avanti 2017.1 version, Optovue Inc., Fremont, CA, USA). Centered at the fovea, the OCTA scanning area was 3 × 3 mm with a resolution of 304 × 304 pixels. The superficial vascular plexus (SVP) and deep vascular plexus (DVP) were divided automatically by the AngioVue software. Cases with segmentation errors were corrected manually. The SVP extends from the inner limiting membrane (ILM) to 9 μm above the inner plexiform layer (IPL)-inner nuclear layer (INL) junction, while the DVP lies between 9 μm above the IPL-INL junction and 9 μm beneath the outer plexiform layer (OPL)-outer nuclear layer (ONL) junction. Two observers (R.G. and R.H.) examined image quality independently and ruled out inferior quality images leading to possible segmentation errors. The whole and parafoveal VD in the SVP and DVP were calculated automatically by the AngioVue software (algorithm version: A2017, 1, 0, 155). Vessel density was defined as the proportion of vessel area with flowing blood over the total measurement area. The whole area was defined as a circle area centered at the fovea with a diameter of 3 mm. The parafoveal area was defined as the whole area minus the central circle area that centered at the fovea with a diameter of 1 mm. Furthermore, FAZ parameters, including FAZ area, FAZ perimeter, acircularity index, and foveal VD in a 300-μm-wide region around the FAZ (FD-300), were measured automatically based on the retina slab (between the ILM and 9 μm beneath the OPL-ONL junction). FAZ perimeter was defined as the length that encompasses the FAZ. The acircularity index was defined as the ratio of the measured FAZ perimeter to the perimeter of a circle with the same area. In addition, foveal thickness was also measured automatically via the built-in software.

### Statistical analysis

SPSS software (version 25.0, IBM Corporation, Chicago, IL, USA) was used for statistical analysis. Before and after adjusting for DR severity and systemic risk factors including sex, age, type of diabetes mellitus, and a history of glycated hemoglobin A1c, hyperlipidemia, hypertension, renal impairment, and smoking [16], the one-way analysis of variance, least significant difference test or Kruskal-Wallis H test were utilized to compare the OCTA metrics among three DME groups with different grades. The univariate and multivariate generalized estimating equations were used to evaluate potential associations between visual acuity and OCTA metrics and other risk factors. A two-tailed *P* value less than 0.05 indicated statistical significance.

## Results

A total of 74 patients (92 eyes) with a mean age of 56.23 ± 12.35 years were included. This cohort was type 2 diabetes-predominant and male-predominant with type 2 diabetes making up 93.24% and males making up 68.92% of the group. The mean level of glycated hemoglobin A1c was 8.12 ± 1.82%, and the mean duration of DM was 13.13 ± 7.67 years. More than half of the participants had hypertension (58.11%), 44.59% of the participants were diagnosed with hyperlipidemia, and 37.84% of the individuals had a history of renal impairment. For DR severity level, 52 eyes had NPDR, while 40 eyes had PDR. This study consisted of 24 eyes with early DME, 53 eyes with advanced DME, and 15 eyes with severe DME. The mean BCVA was 0.2 ± 0.15, 0.48 ± 0.23, and 1.06 ± 0.27 logMAR for early, advanced, and severe DME. LogMAR visual acuity was significantly different among different DME grades (*P* < 0.001).

OCTA data among different stages of DME are displayed in Table 1. After adjusting for DR severity and systemic risk factors, the acircularity index was significantly different among different DME grades (*P* = 0.016), and the increase in the acircularity index was shown in severe DME compared to early (*P* = 0.006) and advanced DME (*P* = 0.003). The whole and parafoveal VD in the DVP in severe DME showed more diffuse vascular rarefaction than early DME (*P* = 0.018, *P* = 0.005, respectively) and advanced DME (*P* = 0.035, *P* = 0.012, respectively). No significant trend in VD in the SVP was found among these groups. Furthermore, foveal thickness was positively associated with DME severity (*P* < 0.001).

**Table 1:** OCTA characteristics among different DME grades

	FAZ parameters				SVP		DVP		
DME severity	FAZ area (mm^2^)	FAZ perimeter (mm)	Acircularity index	FD-300 (%)	Whole VD (%)	Parafoveal VD (%)	Whole VD (%)	Parafoveal VD (%)	Foveal thickness (μm)
Group I Early (n = 24)	0.34 (0.22, 0.39)	2.43 (1.94, 2.77)	1.17 (1.14, 1.25)	39.36 ± 5.58	35.74 ± 4.18	37.23 ± 4.86	41.71 ± 4.98	43.88 ± 5.54	320.00 ± 43.04
Group II Advanced (n = 53)	0.28 (0.20, 0.38)	2.32 (1.92, 2.66)	1.18 (1.15, 1.27)	40.59 ± 4.54	33.88 ± 4.39	35.26 ± 4.89	40.80 ± 5.35	42.91 ± 5.95	451.34 ± 88.39
Group III Severe (n = 15)	0.30 (0.22, 0.46)	2.41 (2.11, 3.48)	1.24 (1.19, 1.55)	41.72 ± 3.95	35.74 ± 5.24	36.67 ± 5.94	37.35 ± 5.86	37.70 ± 7.19	540.07 ± 220.79
*P* value^*^	0.721	0.395	0.005	0.308	0.149	0.251	0.041	0.006	< 0.001
*P* value I vs. II			> 0.999				> 0.999	0.515	< 0.001
*P* value II vs. III			0.007				0.030	0.004	< 0.001
*P* value I vs. III			0.009				0.015	0.003	< 0.001
*P* value (adjusted) ^*†^	0.721	0.856	0.016	0.143	0.809	0.710	0.023	0.007	< 0.001
*P* value I vs. II (adjusted) ^†^			0.954				0.337	0.407	< 0.001
*P* value II vs. III (adjusted) ^†^			0.003				0.035	0.012	< 0.001
*P* value I vs. III (adjusted) ^†^			0.006				0.018	0.005	< 0.001

*OCTA* = optical coherence tomography angiography; *DME* = diabetic macular edema; *FAZ* = foveal avascular zone; *FD-300* = foveal vessel density in a 300-μm-wide region around FAZ; *VD* = vessel density; *SVP* = superficial vascular plexus; *DVP* = deep vascular plexus

*Comparisons among different severity levels of DME

^†^Adjusted for sex, age, type of diabetes mellitus, diabetic retinopathy severity level, and a history of glycated hemoglobin A1c, hyperlipidemia, hypertension, renal impairment, and smoking

**Fig. 1:**
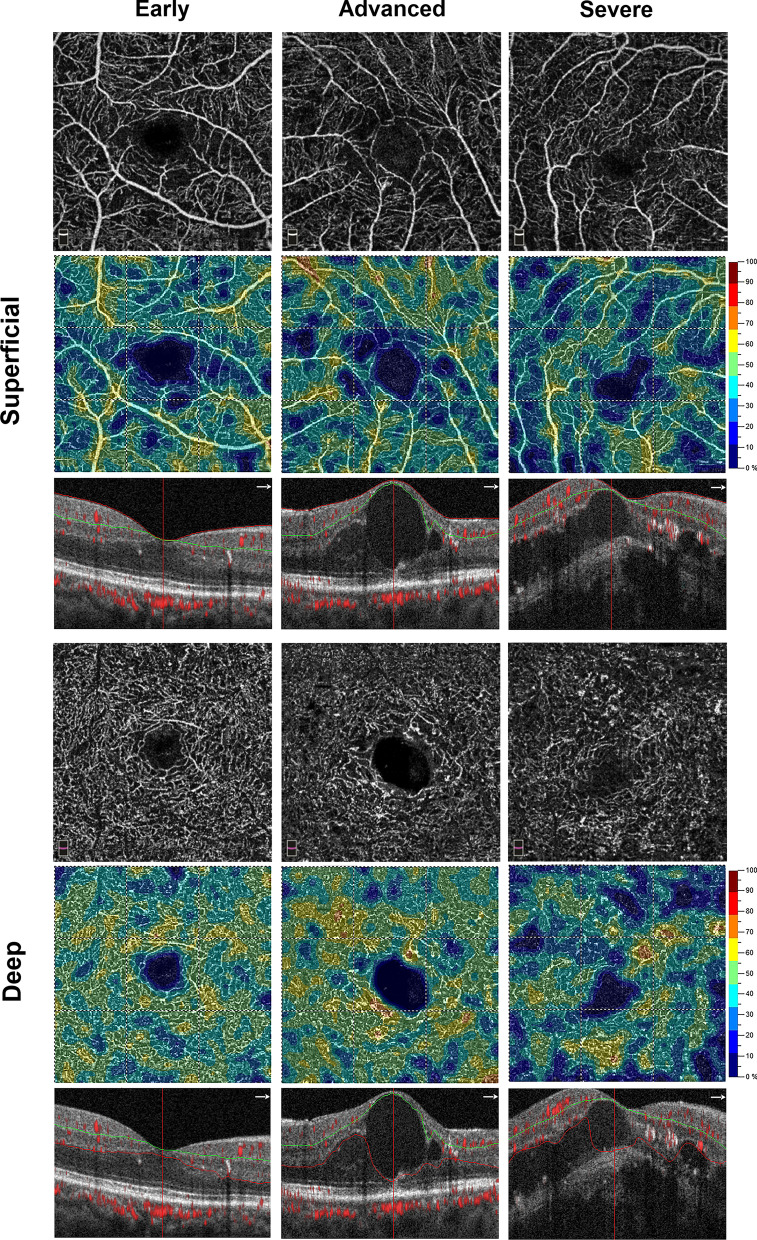
Optical coherence tomography angiography images of superficial vascular plexus (SVP) and deep vascular plexus (DVP) in different stages of diabetic macular edema (DME). As DME progressed, whole vessel density (VD) in the DVP decreased dramatically, while the change in whole VD in the SVP was marginal. In this figure, the eye with early DME has a VD of 38.4% in the SVP and a VD of 44.1% in the DVP, the eye with advanced DME has a VD of 39% in the SVP and a VD of 44.7% in the DVP, and the eye with severe DME has a VD of 36.1% in the SVP and a VD of 35.6% in the DVP

**Fig. 2:**
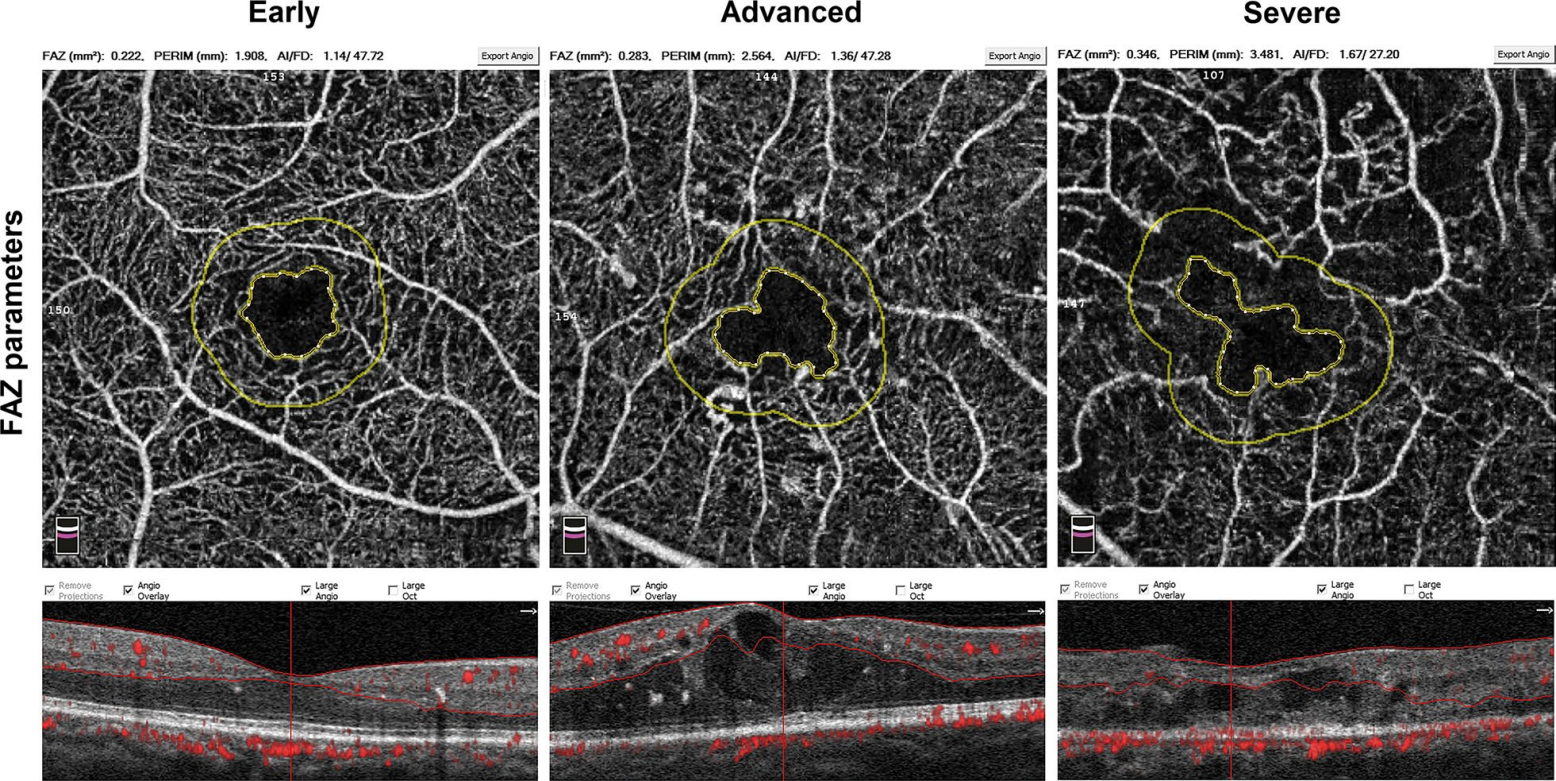
Optical coherence tomography angiography images of foveal avascular zone (FAZ) parameters among different diabetic macular edema (DME) grades. The acircularity index was significantly increased as DME progresses. The eye with early DME has an acircularity index of 1.14, the eye with advanced DME has an acircularity index of 1.36, and the eye with severe DME has an acircularity index of 1.67

Figure 1 presents OCTA images in the SVP and DVP among different DME grades. Figure 2 shows FAZ parameters among different DME grades. Morphologically, severe DME had lower VD in the DVP and higher acircularity index.

Table 2 presents the univariate and multivariate generalized estimating equations of the associations between logMAR visual acuity and OCTA parameters as well as other associated risk factors. For the multivariate generalized estimating equation, a 1 mm^2^ increase in FAZ area was associated with + 0.24 increase in logMAR visual acuity (*P* = 0.007), and a 100 μm increase in foveal thickness was associated with + 0.109 increase in logMAR visual acuity (*P* = 0.001). Moreover, DME severity was associated with logMAR visual acuity (compared to early DME, advanced or severe DME was associated with + 0.223 increase in logMAR visual acuity, *P* = 0.001).

**Table 2:** Univariate and multivariate generalized estimating equations for OCTA parameters and other associated factors with logMAR BCVA in DME

Variables	Univariate		Multivariate	
	β (95% CI)	*P* value	β (95% CI)	*P* value
Sex*	0.060 (− 0.105, 0.224)	0.479		
Age (years)	0.002 (− 0.004, 0.008)	0.458		
DM type^†^	− 0.052 (− 0.224, 0.119)	0.550		
DM duration (years)	0.007 (− 0.004, 0.018)	0.216		
Hemoglobin A1c^‡^	− 0.083 (− 0.238, 0.071)	0.292		
DR severity^§^	0.054 (− 0.105, 0.214)	0.503		
DME severity^||^	0.406 (0.305, 0.507)	< 0.001	0.223 (0.092, 0.354)	0.001
FAZ area (mm^2^)	0.381 (0.160, 0.601)	0.001	0.240 (0.065, 0.415)	0.007
FAZ perimeter (mm)	0.099 (0.039, 0.158)	0.001	− 0.111 (− 0.448, 0.226)	0.519
Acircularity index^¶^	0.068 (0.014, 0.123)	0.013	0.050 (− 0.006, 0.106)	0.081
FD-300 (%)	− 0.002 (− 0.015, 0.012)	0.819		
Whole VD in SVP (%)	− 0.004 (− 0.019, 0.012)	0.640		
Parafoveal VD in SVP (%)	− 0.004 (− 0.019, 0.010)	0.539		
Whole VD in DVP (%)	− 0.010 (− 0.023, 0.004)	0.154		
Parafoveal VD in DVP (%)	− 0.010 (− 0.022, 0.001)	0.085	− 0.002 (− 0.011, − 0.008)	0.711
Foveal thickness (100 μm)	0.140 (0.088, 0.192)	< 0.001	0.109 (0.046, 0.171)	0.001

*OCTA* = optical coherence tomography angiography; *logMAR* = logarithm of the minimum angle of resolution; *BCVA* = best-corrected visual acuity; *DME* = diabetic macular edema; *DM* = diabetes mellitus; *DR* = diabetic retinopathy; *FAZ* = foveal avascular zone; *FD-300* = foveal vessel density in a 300-μm-wide region around FAZ; *VD* = vessel density; *SVP* = superficial vascular plexus; *DVP* = deep vascular plexus; *PDR* = proliferative diabetic retinopathy; *NPDR* = non-proliferative diabetic retinopathy

*Female vs. male

^†^Type 2 DM vs. type 1 DM

^‡^Glycated hemoglobin A1c ≥ 8% vs. glycated hemoglobin A1c < 8%

^§^Eyes with PDR vs. eyes with NPDR

||Eyes with advanced or severe DME vs. eyes with early DME

^¶^Per + 0.1 increase in acircularity index

## Discussion

Recently, an OCT-based grading system called “TCED-HFV” was proposed to assess DME severity. OCTA allows the presentation of different retinal vascular layers. Therefore, it may help to uncover the potential pathology of DME progression. In this cross-sectional observational study, OCTA characteristics among different stages of DME were investigated, and the results suggested that VD in the DVP rather than in the SVP decreased significantly as DME progressed. Furthermore, DME severity, FAZ acircularity index, and foveal thickness were associated with visual acuity. To our knowledge, this is the first study to evaluate associations between OCTA characteristics and DME stages by an OCT-based grading system.

Compared to eyes without DME, macular ischemia was more profound in the DVP of eyes with DME [17, 18]. Congruently, our data also demonstrated that DME severity was associated with decreased VD in the DVP but not with that in the SVP. On the one hand, the decreased VD in the DVP might result in the progression of DME. Fluid production may originate from the SVP, whereas Müller cells and the DVP play roles in fluid removal [19]. We hypothesize that the decreased or absent flow in the DVP places the burden of fluid removal on the Müller cells, consequently contributing to edema at that site due to the imbalance between fluid entry and efflux [20].

On the other hand, it could be that microvascular changes due to the progression of DME occur earlier in the DVP than in the SVP. First, according to the grading of DME severity in the current study [10], a larger intraretinal cyst size partially represents increased DME severity. The capillary segments in the DVP are all interlocked and are approximately 150 μm in length [21], whereas the diameter of the intraretinal cysts could be hundreds of microns [19]. We speculate that the capillaries in the DVP are stretched and interrupted by the increased size of the intraretinal cysts. In addition, inflammation plays an indispensable role in the pathogenesis of DME and results in leukostasis, which is involved in vascular remodeling and capillary nonperfusion [22–25]. Very small vessels, which exist in the entire DVP but only a small part of the SVP, could be plugged by leukocytes [19]. Consequently, the occurrence of leukostasis in the DVP could be more frequent. Finally, the elevated level of vascular endothelial growth factor (VEGF) leads to the development of DME [26]. VEGF has been shown to cause intravascular endothelial proliferation in vivo [27]. Similar to the pathophysiological mechanism of leukostasis, the DVP is more vulnerable to VEGF-induced intravascular endothelial proliferation than the SVP. Hence, the decreased flow of DVP could be positively associated with DME severity.

The association between FAZ area and DME remains to be elucidated. A study showed that the FAZ area was increased in eyes with DME compared to eyes without DME [18]. In contrast, Tarassoly and colleagues confirmed that the FAZ area was equal in diabetic eyes with and without cystoid edema [4]. In this study, both the FAZ area and perimeter were not significantly changed among different DME grades. However, similar to studies suggesting that worsening stages of DR was correlated with higher irregularity in the FAZ [28–30], the acircularity index significantly increased as DME progressed. The possible explanations are as follows. First, there are considerable variations of FAZ metrics in healthy individuals. The FAZ area and perimeter increased with age, and the FAZ area was associated with sex, while the acircularity index, an indicator of irregularity in the FAZ, was independent of age and sex [31]. Second, unlike the FAZ area and perimeter that could be imprecisely measured owing to the alterations in retinal magnification after lens implantation and the difference in axial length, the acircularity index is a quotient that does not require the correction of axial length [28]. Additionally, vascular layer segmentation and the method of calculating FAZ parameters could be responsible for the discrepancy. More importantly, the potential pathophysiology of the increased acircularity index as DME progressed was multifactorial. Mechanical stretch and damage to capillaries due to DME in the foveal region may lead to irregular FAZ development [19]. Foveal neovascularization and capillary abnormalities could also cause irregularity in the FAZ [28, 32]. Therefore, we think the acircularity index could be more sensitive to the progression of DME than the FAZ area and perimeter.

It has been shown previously that the FAZ area, foveal thickness, DRIL, and ELM and EZ disruption are associated with worse visual acuity in DME [18, 33–37]. In our study, advanced and severe DME were characterized by greater foveal thickness, the occurrence of DRIL and/or the disruption or absence of ELM/EZ compared to early DME. These studies mentioned above are in line with our data demonstrating that the FAZ area, foveal thickness, and DME severity were associated with visual acuity in DME.

There are several limitations in this study. First, the presence of DME interferes with OCTA signal intensity [38] and quantitative vascular measurements [39]. Second, the distribution of different DME grades was uneven in this study, and the cross-sectional design of this small-scale study restrained us from assessing the temporal patterns in retinal blood vessels during the progression of DME. Our findings need to be fully validated by a larger, evenly distributed population of DME of a prospective design. In addition, the 3 × 3 mm OCTA scanning field can present only a small area of the retinal posterior pole. Evaluating the OCTA parameters of the perifoveal and peripheral retina is also beneficial for understanding microvasculature variations with the development of DME.

## Conclusion

The progression of DME causes vascular rarefaction in the DVP. Increased DME severity, greater foveal thickness, and larger FAZ area result in worse visual acuity. OCTA could be a promising device for evaluating DME severity and visual acuity.

## Data Availability

The datasets used and/or analyzed during the current study are available from the corresponding author upon reasonable request.

## Abbreviations

BCVA: Best-corrected visual acuity
CRT: Central retinal thickness
DME: Diabetic macular edema
DR: Diabetic retinopathy
DRIL: Disorganization of the inner retinal layers
DVP: Deep vascular plexus
ELM: External limiting membrane
EZ: Ellipsoid zone
FAZ: Foveal avascular zone
FD-300: Foveal VD in a 300-μm-wide region around the FAZ
ILM: Limiting membrane
INL: Inner nuclear layer
IPL: Inner plexiform layer
NPDR: Non-proliferative diabetic retinopathy
OCTA: Optical coherence tomography angiography
ONL: Outer nuclear layer
OPL: Outer plexiform layer
PDR: Proliferative diabetic retinopathy
SD-OCT: Spectral domain optical coherence tomography
SVP: Superficial vascular plexus
VD: Vessel density
